# Supplementary material for: HIVprotI: an integrated web based platform for prediction and design of HIV proteins inhibitors
Source: J Cheminform. 2018 Mar 9;10:12. doi: 10.1186/s13321-018-0266-y (PMC5845081; doi:10.1186/s13321-018-0266-y)
Supplement: Supplementary file 2 — Additional file 2. Source code of HIVProtI web server. [file 13321_2018_266_MOESM2_ESM.zip › HIVprotI_Source-code/hivprotI/weka-3-6-6/doc/weka/gui/boundaryvisualizer/RemoteResult.html]

RemoteResult


---


|  |  |  |
| --- | --- | --- |
| **Overview** \| **Package** \| **Class** \| **Tree** \| **Deprecated** \| **Index** \| **Help** \| **Weka's home** | |  |
| **PREV CLASS**   NEXT CLASS | **FRAMES**    **NO FRAMES**     **All Classes** |  |
| SUMMARY: NESTED \| FIELD \| CONSTR \| METHOD | DETAIL: FIELD \| CONSTR \| METHOD |  |


---


## weka.gui.boundaryvisualizer Class RemoteResult

```
java.lang.Object
  weka.gui.boundaryvisualizer.RemoteResult
```

**All Implemented Interfaces:**: java.io.Serializable

---

``` public class RemoteResult extends java.lang.Object implements java.io.Serializable ```

Class that encapsulates a result (and progress info) for part
of a distributed boundary visualization. The result of a sub-task
is the probabilities necessary to display one row of the final
visualization.

**Since:**
:   1.0

**Version:**
:   $Revision: 1.3 $

**Author:**
:   Mark Hall

**See Also:**: `Serializable`, Serialized Form

---

**Constructor Summary**

`RemoteResult(int rowNum,
int rowLength)`
  
          Creates a new `RemoteResult` instance.


| **Method Summary** | |
| --- | --- |
| `int` | `getPercentCompleted()`             Return the progress for this row |
| `double[][]` | `getProbabilities()`             Return the probability distributions for this row in the visualization |
| `void` | `setLocationProbs(int index, double[] distribution)`             Store the classifier's distribution for a particular pixel in the visualization |
| `void` | `setPercentCompleted(int pc)`             Set the progress for this row so far |

**Methods inherited from class java.lang.Object**

`equals, getClass, hashCode, notify, notifyAll, toString, wait, wait, wait`

**Constructor Detail**

### RemoteResult

```
public RemoteResult(int rowNum,
                    int rowLength)
```

:   Creates a new `RemoteResult` instance.

    **Parameters:**: `rowNum` - the row number: `rowLength` - the number of pixels in the row


**Method Detail**

### setLocationProbs

```
public void setLocationProbs(int index,
                             double[] distribution)
```

:   Store the classifier's distribution for a particular pixel in the
    visualization

    :   **Parameters:**: `index` - the pixel: `distribution` - the probability distribution from the classifier

---


### getProbabilities

```
public double[][] getProbabilities()
```

:   Return the probability distributions for this row in the visualization

    :   **Returns:**: the probability distributions

---


### setPercentCompleted

```
public void setPercentCompleted(int pc)
```

:   Set the progress for this row so far

    :   **Parameters:**: `pc` - a percent completed value

---


### getPercentCompleted

```
public int getPercentCompleted()
```

:   Return the progress for this row

    :   **Returns:**: a percent completed value


---


|  |  |  |
| --- | --- | --- |
| **Overview** \| **Package** \| **Class** \| **Tree** \| **Deprecated** \| **Index** \| **Help** \| **Weka's home** | |  |
| **PREV CLASS**   NEXT CLASS | **FRAMES**    **NO FRAMES**     **All Classes** |  |
| SUMMARY: NESTED \| FIELD \| CONSTR \| METHOD | DETAIL: FIELD \| CONSTR \| METHOD |  |


---
